# What do young people think about their school-based sex and relationship education? A qualitative synthesis of young people's views and experiences

**DOI:** 10.1136/bmjopen-2016-011329

**Published:** 2016-09-01

**Authors:** Pandora Pound, Rebecca Langford, Rona Campbell

**Affiliations:** School of Social and Community Medicine, University of Bristol, Bristol, UK

**Keywords:** sex and relationship education, QUALITATIVE RESEARCH, Meta-ethnography, Young people, sexual health

## Abstract

**Objectives:**

Although sex and relationship education (SRE) represents a key strand in policies to safeguard young people and improve their sexual health, it currently lacks statutory status, government guidance is outdated and a third of UK schools has poor-quality SRE. We aimed to investigate whether current provision meets young people's needs.

**Design:**

Synthesis of qualitative studies of young people's views of their school-based SRE.

**Setting:**

Eligible studies originated from the UK, Ireland, the USA, Australia, New Zealand, Canada, Japan, Iran, Brazil and Sweden.

**Participants:**

Studies of students aged 4–19 in full-time education, young adults ≤19 (not necessarily in full-time education) or adults ≤25 if recalling their experiences of school-based SRE.

**Results:**

–69 publications were identified, with 55 remaining after quality appraisal (representing 48 studies). The synthesis found that although sex is a potent and potentially embarrassing topic, schools appear reluctant to acknowledge this and attempt to teach SRE in the same way as other subjects. Young people report feeling vulnerable in SRE, with young men anxious to conceal sexual ignorance and young women risking sexual harassment if they participate. Schools appear to have difficulty accepting that some young people are sexually active, leading to SRE that is out of touch with many young people's lives. Young people report that SRE can be negative, gendered and heterosexist. They expressed dislike of their own teachers delivering SRE due to blurred boundaries, lack of anonymity, embarrassment and poor training.

**Conclusions:**

SRE should be ‘sex-positive’ and delivered by experts who maintain clear boundaries with students. Schools should acknowledge that sex is a special subject with unique challenges, as well as the fact and range of young people's sexual activity, otherwise young people will continue to disengage from SRE and opportunities for safeguarding and improving their sexual health will be reduced.

**Strengths and limitations of this study**

- The search strategy was comprehensive, using a wide range of manual searches in addition to electronic databases.
- The study includes all eligible qualitative studies of young people's views of their school-based sex and relationship education from around the world for the period 1990–2015.
- The methodology includes measures to ensure robustness and reliability, including double data extraction and the independent screening of publications and quality appraisal by two different researchers.
- The meta-ethnographic method allowed us to provide a fresh interpretation of the evidence, enabling the field to progress and pinpointing areas for policy change. Topics such as sexting and online safety are now discussed within some SRE programmes, but research into young people's views of such programmes had not been published at the time of our synthesis, creating a gap in the evidence.

## Introduction

Sex and relationship education (SRE) in schools remains a fiercely debated topic.^1^ Despite evidence that the reverse is true,^2^ ^3^ some commentators believe that teaching young people about sex and relationships will encourage sexual activity.^4^ On the other hand, those advocating SRE argue that it gives young people the information and skills they need to have safe, fulfilling relationships and to take responsibility for their sexual health.^5^ In England, it is mandatory to teach anatomy, puberty and the biological aspects of sexual reproduction to primary and secondary school pupils, and to teach about sexually transmitted infections (STIs) and HIV/AIDS to pupils in maintained secondary schools. All other elements of SRE are, however, non-statutory in England.^6^ ^7^ The situation varies across the UK, with no statutory requirement at all in Scotland.^7^ In the USA, sex education may be taught as an ‘abstinence-only’ programme or as a ‘comprehensive’ programme (which emphasises abstinence but includes information on safer sex and contraception).^2^ Some states dictate what should be taught while others leave the decision to individual schools. As in the UK and elsewhere, what students actually learn varies widely.

The existing government UK guidance on SRE dates from 2000,^8^ but due to advances in digital technology, young people now occupy a radically different social and sexual landscape. The quality of SRE varies widely, with more than a third of UK schools lacking good-quality SRE in 2013.^9^ However, despite its lack of status and variable quality, school-based SRE is seen as vital for safeguarding young people and for helping to combat child sexual abuse^10^ and exploitation.^11^ It also represents a key strand in policies to improve sexual health outcomes among young people.^12^ Although UK teenage pregnancy rates have come down, they remain high compared with other European countries,^13^ as do UK rates of STIs,^14^ with 16–24 years old accounting for most new diagnoses.^15^ Fortunately there is an association between receipt of school-based sex education and lower reporting of negative sexual health outcomes,^16^ as well as a strong association between unplanned pregnancy and receipt of sex education from sources other than school,^17^ suggesting SRE's ability to positively affect health outcomes. Additionally there is some evidence that SRE may have potential to positively affect academic attainment.^18^ ^19^ Furthermore, although it seems counterintuitive given the availability of online material, young people want SRE to be taught in schools^20^ ^21^ and increasingly cite school as their main source of sexual health information.^22^

An understanding of young people's views is essential if high-quality SRE programmes are to be developed.^6^ Young people consistently report in surveys that SRE starts too late, is too biological, negative, insufficiently comprehensive and poorly delivered.^23–26^ However, surveys cannot provide the depth of understanding necessary if fundamental improvements are to be made. To obtain greater insight into young people's perspectives, we conducted a synthesis of qualitative studies of their views and experiences of their school-based SRE. This study formed part of a multicentre collaboration funded by the UK National Institute for Health Research (School for Public Health Research) to develop a sexual health and alcohol intervention for young people in schools.

## Methods

We have adhered to guidelines for enhancing transparency in reporting qualitative syntheses^27^ (see online supplementary material).

10.1136/bmjopen-2016-011329.supp1 supplementary appendices

### Eligibility

As the wider collaboration was concerned with developing a sexual health and alcohol intervention, we originally aimed to identify studies of programmes that combined SRE with alcohol education, but excluded those that focused solely on alcohol education. Studies that focused solely on HIV/AIDS prevention were also excluded, as were those involving special schools or students with special needs. Programmes had to be either school based or linked to schools. Studies were included if samples were of students aged 4–19 in full-time education, young adults (not necessarily in full-time education) if most were under 19 or adults no older than 25 if recalling their education. Studies had to involve qualitative methods of data collection and analysis. If studies using mixed methods included a qualitative element that met our criteria, this element was included. Since attitudes towards sexual health have changed considerably over the past few decades, we limited our search to studies conducted in or after 1990, envisaging that those conducted before 1990 would be of little relevance today. Within the context of the databases searched, eligibility was not restricted by language or country.

### Searches

**Figure 1:**
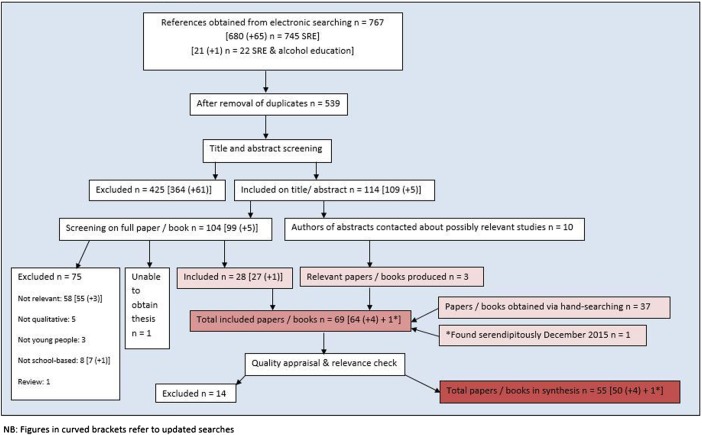
Flow chart of search and appraisal process. Figures in curved brackets refer to updated searches. SRE, sex and relationship education.

We searched MEDLINE, Embase, PsychInfo, Sociological Abstracts, IBSS, ERIC, Web of Science and Cinahl using simple searches (table 1), having previously found these as effective for locating qualitative studies as complex searches.^28^ ^29^ We included additional searches to specifically target studies combining SRE with alcohol education. References were screened on title and abstract, with a random sample (35%) being independently screened by a second reviewer (84% agreement). One hundred and nine were selected as potentially relevant, including 10 conference abstracts whose authors were contacted. Full publications were obtained for the remaining 99, of which 86 (8 first needed translating, 5 were difficult to obtain) were screened by 2 independent reviewers (84% agreement). We hand-searched key journals identified by colleagues from 1990 onwards and searched the websites of relevant agencies. We contacted key authors, checked references of relevant papers and sought books in the University of Bristol libraries and using internet search engines (table 2). Searches were conducted from March to May 2014 and updated in February 2015. A further publication was identified serendipitously in December 2015 and incorporated into the synthesis. References were managed on Eppi-Reviewer V.4.^30^ The search process is shown in figure 1.

**Table 1:** Search terms for electronic databases

	MEDLINE, Embase, PsychInfo	Sociological Abstracts, IBSS, ERIC, Web of Science, Cinahl
SRE	Adolescent/ young people.mp. 1 or 2 (sex and relationships education).mp. Sex Education/or sexual health education.mp. 4 or 5 Qualitative Research/ qualitative.mp. 7 or 8 3 and 6 and 9 10 and 1990:2014.(sa year)	(adolescent* or child*) AND (view* or perception* or experience*) AND (‘sex education’ or SRE or ‘sexual health education’) AND (school*) AND (qualitative)
SRE and alcohol education	(sex and relationships education).mp. Sex Education/ sexual health education.mp. alcohol education.mp. 1 or 2 or 3 4 and 5	(adolescent* or child*) AND (view* or perception* or experience*) AND (‘sex education’ or SRE or ‘sexual health education’) AND (alcohol) AND (school*) AND (qualitative)

SRE, sex and relationship education.

**Table 2:** Hand-searching and results

Hand-searching method	Details of hand-search	Results of hand-search
Hand-search of journals	Sex Education; Gender and Education; Culture, Health and Sexuality; British Journal of School Nursing; American Journal of Sexuality Education; Journal of School Health; Sexualities; Health Education Research; Health Education; Health Education Journal; Health Education and Behaviour; Journal of Adolescent Health	15
Checking references	Reference lists of all relevant papers	12
Key authors/contacts	Authors of key papers/key contacts in field	6
Hand-search of websites	Brook, Family Planning Association, Girl Guides, Joseph Rowntree, YWCA, YMCA, Action for Children, Nuffield Foundation, Sex Education Forum, UK Youth Parliament and RSE Hub	3
Libraries	Education Library and Arts and Social Sciences Library (University of Bristol)	1
Internet searches	Google Scholar, Amazon	0
Total		37

### Quality appraisal

We reviewed 10 checklists for appraising qualitative research to derive our quality criteria (see online supplementary appendix 1). Included publications were independently appraised by two reviewers (the first and second authors) and the results then compared.

### Data extraction

Text was extracted from the results and discussion sections of publications and stored on Excel. The first author extracted young people's views and experiences (first-order constructs)^31^ from all papers remaining after quality appraisal. The second author then independently extracted first-order constructs from a random sample of 50% of the papers, finding excellent (non-statistical) agreement. Both then independently extracted authors' interpretations of young people's views and experiences (second-order constructs)^28^ ^29^ ^31^ ^32^ where these were present, before discussing and agreeing these for each publication.

### Synthesis methodology

The first author analysed the data using a combination of meta-ethnography and thematic synthesis.^33^ Taking an inductive approach she first organised the second-order constructs into four broad topics (how SRE is taught, who teaches it, SRE content and gender) and then systematically organised first-order constructs for each paper into the second-order constructs within each topic. First-order constructs that had not been interpreted by authors of primary studies were analysed to produce new second-order constructs. This process produced a basic synthesis for each of the four topics. She then combined the four syntheses into one by further interpreting the data to develop a ‘line of argument’,^34^ in the process creating third-order constructs^31^ (interpretations of first-order and second-order constructs). All authors agreed on the third-order constructs and line of argument. Second-order and third-order constructs are given in table 3.

**Table 3:** Second-order and third-order constructs

	Authors
Third-order constructs	
*Schools have not sufficiently acknowledged that sex is a powerful, intimate, potentially embarrassing and anxiety provoking subject* (overarching third-order construct)	Pound *et al* 2016, present paper
Discussing sex within school renders students and teachers vulnerable	
Since sex is such a powerful subject, SRE defuses it by presenting it as a scientific topic	
Since sex is such a powerful subject, SRE contains it within a moral framework	
*Schools have difficulty accepting young people's sexual activity* (overarching third-order construct)	
Because of the difficulty of accepting young people's sexual activity, SRE is out of touch with young people's lives	
Because of the difficulty of accepting young people's sexual activity, SRE fails to discuss issues relevant to sexually active young people	
Second-order constructs	
The assumption inherent in SRE, that sex is not embarrassing, is insensitive to young people	van Teijlingen *et al*^37^
SRE is a potentially threatening subject, creating anxieties about other students’ reactions, especially those of the opposite sex; discomfort manifests in a reluctance to ask questions and sometimes disruptive behaviour	Buston *et al*^38^
Young men put on a performance of masculinity, conforming to stereotypical cultural values	Measor *et al*^42^
Young men’ behaviour in sex education classes conforms to social codes that valorise masculine resistance to authority	Chambers *et al*^45^
Exposure of lack of knowledge will entail loss of face and shame for young men; knowing what to do in a sexual encounter is defined as male responsibility	Measor *et al*^42^
Young men cannot engage in SRE because to ask for information reveals their existing lack of knowledge in a context where the masculine ideal is to be proficient and experienced	Limmer^46^
Male sexuality is about owning and controlling sexual power and agency and because therefore young men cannot acknowledge a lack of sexual knowledge and have to come across as sexually competent	Chambers *et al*^45^
Young men are potentially vulnerable in SRE lessons where admitting a lack of sexual knowledge might threaten their masculinity	Hilton^44^
Possession of sexual knowledge is linked to status within student peer groups	Thomson and Scott^48^
Claims to sexual knowledge and expertise have a role in achieving a place at the top of the male hierarchy; young men’ disruptive behaviour represents an attempt to take control in the classroom to prevent exposure of their lack of sexual knowledge	Measor *et al*^42^
Young women are vulnerable in mixed-sex education classes because participation in the lesson can be used by young men to attack their sexual reputations	Strange *et al*^47^
Exposure of ‘too much’ knowledge by young women led to verbal attacks by young men on young women; young men’ behaviour in SRE classes rendered the young women almost invisible in the classroom	Measor *et al*^42^
Teacher as protector (and friend) reduces student discomfort; trust between pupils reduces student discomfort; fun reduces student discomfort in SRE	Buston *et al*^38^
Young people believe that scientific information does not articulate with everyday practice	McKee *et al*^71^
Sex education that presents sexual activity as clinical or scientific de-eroticises and disembodies it	Allen^67^
The forms of knowledge that seem to feature in SRE are those prescribing appropriate behaviour	Allen^72^
Young people learn from school and parents that sex is bad and you should neither have sex nor prepare for it	McKee *et al*^71^
SRE presents a ‘legitimate’ model of sexuality, its power lying in what it omits rather than what it includes	Thomson and Scott^48^
Invisibility of homosexuality denies the possibility of discussing sex or emotions within same-sex relationships	Allen^67^
Young gay men may be less inclined to pay attention to SRE that only addresses heterosexual interactions	Kubicek *et al*^78^
SRE messages are gendered and reproduce gender inequalities	Castro-Vasquez and Kishi;^76^ Levin^61^
SRE insists on young women taking responsibility for ‘doing the work’ of sexual relationships	Chambers *et al*^45^
SRE places young women in the role of sexual gatekeeper	Levin^61^
SRE messages give young women the message that their sexual desires are mild compared to those of young men	DiCenso *et al*^57^
Traditional SRE leaves no space for discussion of female pleasure	Hirst^79^
SRE reproduces sexist perceptions of women as lacking in desire and agency; participants’ descriptions of their sexual activity run counter to normative constructs of female behaviour and to the content of much sex education	Hirst^80^
Refusal to include non-reproductive aspects of sexuality reinforces a passive and negative view of female sexuality; SRE's reproductive paradigm shapes young women's understandings of what is normal and acceptable	Thomson and Scott^48^
The informal sexual subculture is separate from the official school culture but that the two cultures collide in SRE	Thomson and Scott^48^
There is dissonance between SRE and the everyday lives of young people	Langille *et al*^51^
Young people's view of themselves as legitimately sexual and interested in achieving positive sexual experiences was not congruent with the school's view of them, possibly explaining their disengagement from SRE	Allen^73^
The ‘discourse of erotics’ was missing from sexuality education; young people prioritise the ‘discourse of erotics’ over the ‘official’ discourse	Allen^72^
The focus on vaginal penetrative sex in SRE undermines young women’ ability to resist this in favour of less risky practices	Hirst^79^
Much of young people's sexual activity is ‘safer sex’ but this not acknowledged in SRE	Hirst^80^
The sorts of risk discussed in SRE seem less real, immediate or meaningful for young men than the immediate risks to them of deviating from performing the approved version of sexualised masculinity	Limmer^46^
Discourses of safer sex were resisted by young people if they threatened a desired sexual identity; SRE pays insufficient attention to the social context in which sexual risk-taking occurs	Abel and Fitzgerald^74^
Some young people's culture is a significant factor in their sexual development and SRE can be insensitive to this	Rawson and Liamputtong^66^
The credibility of sex educators was linked to trust and confidentiality	Kimmel *et al*^39^
Delivery of SRE by teachers that are known to students has the potential to disrupt existing relationships and breach boundaries; delivery of SRE by teachers commonly invoked concerns about breaches of confidentiality; pupils want privacy for their feelings	Alldred and David^20^
Where sexual issues are not dealt with openly in school young people regard sexual matters as something ‘naughty’	Woodcock *et al*^41^
Young people learn that you should not talk about sex	Mckee *et al*^71^
The perception of SRE teachers as ‘old’ creates a fear of being judged about their sexual behaviour	Langille *et al*^51^
The teacher–student relationship with its power relations provides a problematic context for discussions of sexuality	Lupton and Tulloch^50^
The parent/child like relationship between teachers and pupils contributes to antagonistic interactions in class; students feel excitement at shifting the balance of power by making a teacher feel vulnerable	Haste^86^
Teachers were put on trial with respect to how they cope with delivering SRE	Woodcock *et al*^41^
Peer educators’ similarity of age and status engender feelings of affinity	Kidger^88^
The looser classroom management in peer-led lessons provides more opportunities for young men to dominate	Forrest *et al*^40^

SRE, sex and relationship education.

## Results

### Searches

Twenty-eight publications were identified electronically, 3 by contacting authors of abstracts identified electronically, 37 by hand-searching and 1 serendipitously, producing 69 publications in total (figure 1).

### Quality appraisal

The quality of publications was generally good except in relation to reporting methods (table 4). Most demonstrated theoretical insight but of those that did not, most were considered relevant and added to the weight of evidence.^29^ Fourteen papers were excluded: six on the grounds of quality and eight for lacking sufficient data on the synthesis topic (see online supplementary appendix 2).

**Table 4:** Quality appraisal for 69 studies

Quality appraisal criteria	Yes	No	Unsure		
Does the research involve qualitative methods of data collection and analysis?	96% (66)	4% (3)			
Does the research have clear aims and objectives?	90% (62)	10% (7)			
Were the data collected in a way that addressed the research aim?	99% (68)	1% (1)			
Was the data analysis sufficiently rigorous to address the aims of the research?	71% (49)	4% (3)	25% (17)		
Can one be confident that all the relevant data were taken into account?	74% (51)	25% (17)	1% (1)		
Were sufficient data presented to support the interpretations made?	91% (63)	9% (6)			
	Good	Acceptable	Poor		
What was the quality of reporting of the methods?	39% (27)	29% (20)	32% (22)		
	Yes	No	No, but useful		
Did the paper demonstrate theoretical insight, novel findings or perspectives?	57% (39)	14% (10)	29% (20)		
Overall quality assessment	Very good	Good	Acceptable	Not very good	Very poor
	3% (2)	30% (21)	48% (33)	17% (12)	1% (1)

### Characteristics of studies and participants

The remaining 55 publications (see online supplementary appendix 3) originate mainly from the UK, followed by the USA and represent 48 studies spanning 25 years (see online supplementary appendix 4). They consist of 51 peer-reviewed journal articles (all of which were in English except for one Portuguese paper), one PhD and three books. We found no studies about combined sexual health and alcohol education. Most were of standard school-based SRE delivered to secondary school pupils by teachers and most collected data using focus groups. Students' ages ranged from 6 to 25, but only one study^35^ was of younger children's views (aged 6–12) and only a minority included participants between the ages of 18–25; the majority of participants were aged 12–18 (see online supplementary appendix 4).

## Synthesis

**Figure 2:**
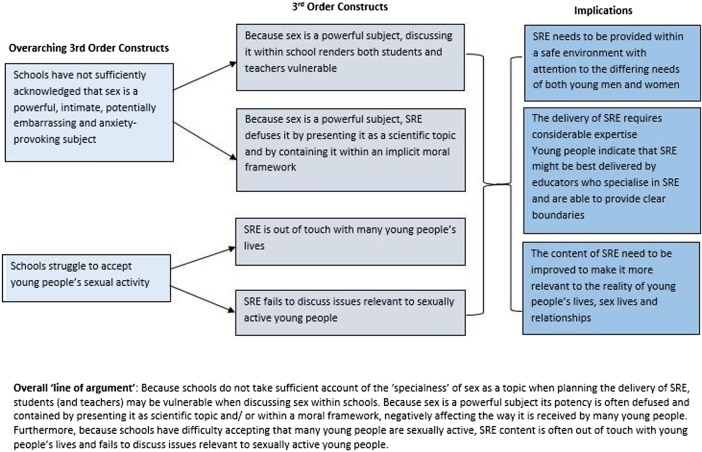
Map of synthesis. *Overall ‘line of argument’*: Since schools do not take sufficient account of the ‘specialness’ of sex as a topic when planning the delivery of SRE, students (and teachers) may be vulnerable when discussing sex within schools. Since sex is a powerful subject, its potency is often defused and contained by presenting it as scientific topic and/or within a moral framework, negatively affecting the way it is received by many young people. Furthermore, because schools have difficulty accepting that many young people are sexually active, SRE content is often out of touch with young people's lives and fails to discuss issues relevant to sexually active young people. SRE, sex and relationship education.

We arrived at two overarching interpretations (third-order constructs) that we felt explained most of the data. First, schools have taken insufficient account of the ‘specialness',^36^ or distinctive nature, of sex as a topic. Sex is a potent subject that can arouse strong emotions, reactions and feelings—of anxiety, embarrassment and vulnerability among others—yet the prevailing approach within schools appears to be to deny that there is anything exceptional about the topic and to attempt to teach SRE in the same way as other subjects. This negatively affects the delivery of SRE and renders young people vulnerable. Second, schools appear to struggle to accept that some young people are sexually active. This leads to SRE content that is out of touch with many young people's lives and a failure to discuss issues relevant to sexually active young people. The remainder of the data consist of young people's views on who should deliver SRE. An overall map of the synthesis is shown in figure 2. Quotes from primary studies typifying young people's views are presented in box 1.

Box 1 **In young people's words**

Discussing sex within school renders students and teachers vulnerable

*Teachers*: ‘I think a lot of teachers are actually embarrassed …’. (ref. 40, p. 207)

Zara: ‘We had Miss Plum she was a PE teacher but she cried’. Zoe: ‘Yeah she did aye.’ Zara: ‘So we just felt sorry for her, [student's name] made her cry about three times’. Zoe: ‘Yeah’. Zara: ‘Like it was so horrible’. (ref. 53, p. 40)

*Young men*: ‘Yeah, some people are too scared to say things so they cover that up by being noisy and disrupt the class’. (ref. 47, p. 206)

*Young women*: ‘Mrs Blackshaw, right, she can't really talk to us properly about it because the boys start making snide remarks and everything like that … so you get to the stage where if you do want to ask anything you won't ask it because the boys will start making remarks’. (ref. 38; p. 325)

Since sex is such a powerful subject SRE defuses it by presenting it as a scientific topic

‘Ah, they never really talked about sex (…) Like the sperm goes up the fallopian tube, hits the egg…we don't care about that’. (ref. 51, p. 250)

‘Everything we got in our class had a really clinical feel, it's just like information but it's not related to yourself’. (ref. 70, p. 103)

Since sex is such a powerful subject SRE contains it within a moral framework

‘All they ever do is talk about the dangers of sex and that, and nothing about the pleasure’. (ref. 42, p. 126)

‘They don't mention anything about same-sex relationships or homophobia, I think they should so more people are aware’. (ref. 77, p. 258)

‘I think that their main focus in on, you don't feel pressured to have sex and things like that. I think that they depict it was the guy wants to have sex and the girl doesn't. And the guy is like, ‘Oh it will feel good’. They don't realise that a lot of girls want to have sex, too. And so I don't think that they focus on that enough at all’. (ref. 69, p. 465)

Because of the difficulty of accepting young people's sexual activity SRE is out of touch with young people's lives

‘We discuss contraception and sex but not what to do when having sex. We don't know’. (ref. 42, p. 100)

‘So you just were taught about sexual intercourse causing pregnancy, but you were never taught about masturbation; you were never taught about oral sex all the different, other types of sexual practices…’. (ref. 75, p. 224).

‘As I started experimenting and everything I was like, I can't believe we were taught that this is not okay’. (ref. 70, p. 103)

‘Didn't really say anything about oral sex, and maybe people want to have oral sex with their partners but are not ready to have sex’. (ref. 42, p. 128)

Because of the difficulty of accepting young people's sexual activity SRE fails to discuss issues relevant to sexually active young people

‘They don't really go into the whole relationships thing partly because I don't think—they don't want us to have relationships’. (ref. 71, p. 657)

‘They didn't talk about the emotional part of having sex. They didn't really talk about how sex will affect you as a person and how it affects your emotions’. (ref. 70, p. 103)

‘They didn't really help you with your sexual feelings, they made you kind of feel bad about having them’. (ref. 70, p. 103)

‘It's not just about what happens when the sperm meets the egg, or putting a video on, I had that at school. They have to talk to young people about all the pressures out there, from the media, from family, friends, all that's important’. (ref. 66, p. 354)

SRE is valued by young people from ethnic minorities and much younger children

‘You get some good information in these classes. All my mother would tell me, she would tell me like myths, hypothetical things, things that old ladies from generation to generation will tell her. Like ′Mom, that's not even true!’’. (ref. 84, p. 263)

Improving SRE

*Teachers*: ‘If your teacher who's a grown up can't talk about it, how are you (supposed to)? That gives you the impression that, oh I'm not really supposed to talk about it’. (ref. 54, p. 450)

‘Not teachers because [teachers] know you, judge you, and they like to talk about you’. (ref. 39, p. 177).

Harry: ‘Not a teacher’. Ginny: ‘For embarrassment reasons and knowledge reasons as well’. Harry: ‘Yeah cause the teachers, the teachers talk so much like’. Odette: ‘And you kind of know them as well before like before’. (ref. 53, p. 40)

PA: ‘Who do you want to learn it (SRE) from?’ Girls (chorus): ‘Somebody we don't know’. Girl: ‘The school nurse or somebody that comes in’. PA: ‘So you all agree? What are the reasons for having somebody you don't know?’ Girl: ‘So when you tell them something they don't know you’. Girl: ‘You don't get embarrassed like in front of teachers’. Girl: ‘Well, you'd be embarrassed the next day with a teacher’. Girl: ‘We don't want them to know all about us’. PA: ‘Is it about privacy?’ Girl: ‘Yeah. It's like your own feelings…it was our form tutor!’. (ref. 20, p. 88)

*Peer educators*: ‘They treated us with respect, they treated us like fellow students instead of … pupils’. (ref. 40, p. 204)

*Outside experts*: ‘She was not ashamed of it, we saw that she thought it was fun to talk about it, that she really thought it was fun, her job’. (ref. 52, p. 121)

‘You want someone who's not from the school or someone who actually does it as a job and knows what they're talking about and you know can be professional about what they are telling you and …’. (ref. 53, p. 41)

### Schools have taken insufficient account of the specialness of sex

Discussing sex within school renders students (and teachers) vulnerable.

Students reported embarrassment and discomfort, particularly in mixed-sex SRE,^37^ which impeded their engagement^38^ as they feared humiliation in front of the class.^39^ ^40^ Young men reported that they were disruptive in SRE,^38^ ^41–47^ with some explaining their behaviour as a way of masking their anxiety.^47^ It was observed that men are expected to be sexually knowledgeable and competent.^42^ ^44–46^ ^48^ ^49^ Young men were reluctant to risk revealing themselves as sexually inexperienced by asking a question;^20^ they were keen to assert that they already knew everything,^41^ ^42^ ^50^ but some wanted teachers to understand their inability to admit to any ignorance about sex.^44^ Consequently, young men are vulnerable in SRE^44^ and their disruptive behaviour may represent an attempt to prevent exposure of sexual ignorance.^42^ Young women are also vulnerable; they were observed to take SRE seriously,^38^ ^42^ ^47^ ^50^ but in mixed classes, young men often discouraged their participation by verbally harassing them^20^ ^40^ ^42^ ^45^ ^47^ ^51^ and attacking their sexual reputations if they engaged in the class.^42^ ^47^ ^52^ Some teachers reportedly failed to confront young men about their behaviour^42^ or were complicit with them.^51^ Teachers themselves were commonly reported to be embarrassed and awkward delivering SRE.^37^ ^39^ ^40^ ^42^ ^48^ ^50–56^

Young people wanted to receive SRE in a safe and confidential environment^43^ ^44^ ^56^ where they could participate uninhibitedly^57^ without being singled out.^58^ They suggested group discussions,^43^ ^44^ ^47^ ^54^ ^56^ ^58^ ^59^ skills-based lessons, demonstrations^56^ ^57^ ^60^ and diverse activities,^43^ ^55^ ^56^ ^61^ ^62^ appreciating dynamic teaching techniques.^63^ Some advocated small group teaching^41^ ^46^ or smaller classes that were easier to control.^44^ Class control was deemed essential for SRE^43^ but was often reported to be lacking.^41^ ^48^ ^53^ Teachers who maintained control and protected students from ridicule increased safety and engagement in SRE, as did building trust between classmates.^38^ Ground rules for discussion, behaviour^51^ ^56^ and confidentiality^58^ were felt to reduce discomfort, as were humour^47^ ^52^ ^57^ and fun.^38^ ^40^ ^64^ Some young women^20^ ^59^ and girls^35^ expressed a preference for single-sex classes. In one study, most young women wanted all or some SRE in single-sex classes, but most young men wanted mixed classes.^47^

Sex is a potent subject, so SRE defuses it by presenting it as a scientific topic.

Young people criticised SRE for being overly biological^20^ ^48^ ^51^ ^58^ ^65^ ^66^ and for presenting sex as a scientific activity,^42^ ^67^ deeming this approach basic,^40^ ^41^ ^48^ ^51^ repetitive,^51^ ^54^ ^68^ narrowly focused,^39^ ^68^ ^69^ technical^69^ ^70^ and above all, irrelevant.^46^ ^48^ ^54^ ^56^ ^63^ ^68^ ^71^ This approach was also observed to de-eroticise and disembody sex.^67^

Sex is a potent subject, so SRE contains it within an implicit moral framework.

SRE was described as prescribing appropriate behaviour,^72^ shaping student sexuality as a ‘problem’ to be managed^73^ and presenting a model of ‘legitimate’ sexuality.^48^ Young people noted a focus on unwanted pregnancy and STIs,^40^ ^42^ ^46^ ^54^ ^57^ ^63^ ^67^ ^70^ ^74^ ^75^ also observing the negative portrayal of teenage pregnancy^67^ and the casting of young men as sexual predators.^40^ ^46^ SRE was described as heteronormative;^61^ ^76^ young people reported that homosexuality was barely mentioned,^48^ ^63^ ^74^ ^77^ ^78^ rendering lesbian, gay, bisexual and transgendered students invisible.^67^ ^77^ ^79^ Students wanted homosexuality to be discussed within SRE^42^ ^44^ ^56^ ^62^ ^63^ ^69^ ^75^ to facilitate discussion of same-sex relationships,^78^ help normalise these relationships, address homophobia and support young lesbians, gay men and bisexuals.^77^ Students further described SRE as gendered,^54^ ^61^ ^63^ ^80^ noting that it failed to discuss female pleasure,^79^ reproduced stereotypes of women as passive^48^ and lacking in desire,^57^ ^69^ ^80^ placed responsibility for the work of sexual relationships onto women^45^ ^57^ and cast them as sexual gatekeepers.^42^ ^48^ ^61^

### Schools struggle to accept that some young people are sexually active

Since schools have difficulty accepting young people's sexuality SRE is out of touch with many young people's lives.

It was observed that young people disengage from sources of information that do not match their own experiences^71^ and certainly there appeared to be a ‘collision’^48^ between SRE and young people's culture.^51^ ^72^ Young people noted that SRE defined sex narrowly as heterosexual intercourse,^42^ ^63^ ^78^ ^80^ failing to acknowledge the full range of sexual activities they engaged in^42^ ^75^ ^78^ ^80^ and that they wanted to discuss,^39^ ^42^ ^55^ ^57^ ^74^ many of which actually constituted ‘safer sex’.^80^ While many young people see themselves as sexual beings (although not necessarily having sexual intercourse), SRE does not support this view.^67^ ^71^ ^73^ Young people wanted SRE to reflect their emotional and sexual maturity,^81^ their autonomy^61^ and for some, the fact of their sexual activity.^42^ Many disliked the emphasis on abstinence,^54^ ^56^ ^70^ ^81^ finding it unrealistic^57^ and contrary to their reality.^74^ With regard to sexual risk-taking, young people's accounts suggest that some perceive the risks of unsafe sex to be less than the risks to identity that safer sex might entail (eg, loss of erection due to discussing condoms during sex).^46^ ^74^ Furthermore, it was suggested that if young people receive the message that sex is wrong, they may also believe that preparing for it is wrong and fail to organise contraception.^71^

Young people reported that SRE was delivered too late^20^ ^41^ ^42^ ^44^ ^54^ ^56^ ^57^ ^60^ ^62^ ^68^ ^80^ and that starting it earlier might make it easier to discuss sex.^41^ ^56^ ^81^ They wanted more openness in SRE,^50^ ^63^ wanting to talk more about sex,^42^ ^51^ what sex involves^72^ and how to have sex.^48^ ^55^ Young men particularly wanted this information,^41^ ^44^ ^47^ ^49^ expressing anxiety about being able to ‘perform’^42^ ^82^ and disappointment that SRE did not help with this.^42^ Many reported turning to pornography instead.^42^ ^44^ ^46^ ^49^ ^76^ ^82^ It was also observed that SRE lacks a ‘discourse of erotics'.^72^ Young people wanted to know how to make sex pleasurable^55^ ^63^ ^67^ ^73^ ^75^ but reported this was not discussed.^70^ ^80^ While some wanted to talk about sexual pleasure in SRE,^63^ others felt this might be too personal.^73^

Since schools have difficulty accepting young people's sexuality, SRE fails to discuss issues relevant to sexually active young people.

SRE failed to provide information that sexually active young people wanted, including on community health services,^57^ the options available if pregnancy occurred^56^ ^67^ ^83^ and the pros and cons of different contraceptive methods (table 5). Also frequently absent were conversations about relationships^39^ ^41^ ^70^ ^71^ ^74^ and the feelings accompanying sexual activity,^42^ ^70^ which young people wanted to discuss.^44^ ^55–57^ ^68^ ^81^ Young women particularly wanted to talk about emotions^42^ ^69^ and relationships.^55^ ^61^ ^67^ ^68^ ^75^ Some young people wanted to learn how to refuse sex,^56^ ^74^ or to discuss sexual abuse^56^ ^75^ or the pressures they experienced.^44^ ^49^ ^66^ Some found the idea of talking straightforwardly about their sexual needs difficult,^71^ but others wanted to learn these skills^55^ ^57^ ^61^ and become more confident in sexual negotiations and discussions.^55^ ^74^

**Table 5:** Information on contraception, STIs, abortion, adoption and pregnancy is not sufficiently comprehensive

SRE does not adequately cover these subjects	Authors
Contraception, pros and cons of different types of contraception	Dicenso *et al*;^57^ Eisenberg *et al*;^56^ Woodcock *et al*;^41^ O'Higgins and Gabhainn^55^
Emergency contraception and its adverse effects	Lester and Allan;^81^ Abel and Fitzgerald^74^
Different opinions on contraceptive pill, adverse effects of contraceptive pill	Measor *et al*^42^
Contraceptives other than the condom	Buston and Wight;^49^ Hilton;^44^ Broadbear and Broadbear;^62^ Limmer^46^
Where to obtain different forms of contraception, how to buy condoms	Abel and Fitzgerald;^74^ Eisenberg *et al*;^56^ O’Higgins and Gabhainn^55^
What to do if no contraception available	O’Higgins and Gabhainn^55^
Why condoms should be used	Kubicek *et al*;^78^ O’Higgins and Gabhainn^55^
How to use male and female condoms; importance of lubrication	Kimmel *et al*^39^
Options if become pregnant, ie, adoption, abortion, teenage pregnancy	Allen;^67^ Eisenberg *et al*;^56^ Measor *et al*^42^
Unbiased information on abortion and how to deal with an abortion	Thomson and Scott;^48^ Rolston *et al*;^75^ Allen;^67^ Eisenberg *et al*^56^
STIs, including transmission through oral sex	Measor *et al*;^42^ Dicenso *et al*;^57^ Lupton and Tulloch;^50^ Hilton;^44^ Kimmel *et al*;^39^ Broadbear and Broadbear 2011; Lester and Allan^81^

SRE, sex and relationship education; STI, sexually transmitted infection.

Nevertheless, SRE was valued by two distinct groups of students. Although it was reported to be occasionally culturally insensitive,^66^ ^80^ ^84^ SRE was appreciated by some students from ethnic or religious minorities.^58^ British-born Chinese students valued it because sex was not discussed within their families,^85^ while several young Muslim women appreciated it for challenging the ‘value-laden’ information they received at home.^84^ Similarly, younger children (6-year to 12-year olds), despite finding some of the content uncomfortable, valued a sexual abuse prevention programme for the skills and information it imparted.^35^ It appeared that these younger children were less reticent (in terms of engaging in the class) and more positive about their SRE delivery than older children.

### Young people's views on SRE educators

While schoolteachers were occasionally praised,^53^ ^54^ ^61^ they were generally regarded as unsuitable for teaching SRE due to lack of training^48^ ^50^ ^51^ ^56^ ^68^ and embarrassment, which was perceived to affect the quality of SRE^51^ ^81^ and to be associated with reliance on passive teaching methods such as outdated films.^41^ ^55–57^ ^81^ Students reported that teachers seemed unable to discuss sex frankly^41^ ^56^ ^81^ and responded unsatisfactorily to questions.^40^ ^42^ ^51^ ^68^ Since credibility was related to the ability to talk frankly about sex,^39^ ^43^ embarrassed teachers were deemed incapable of teaching SRE.^56^ Students found it difficult being taught by such teachers,^55^ suggesting that they diminished their enjoyment of lessons^40^ and gave the impression that sex could not be discussed straightforwardly.^41^ ^54^ ^71^ ^76^

Furthermore, while most believe that ideally teachers should deliver SRE because they know their students, it was this very familiarity that many students found inappropriate, provoking suggestion that delivery of SRE by a familiar teacher has potential to disrupt existing relationships and breach boundaries.^20^ Some students described their embarrassment at discussing sexual and personal matters with teachers they knew^20^ and found it awkward seeing teachers around school afterwards.^52^ For some, the fact of a subject teacher speaking about sex or relationships was regarded as ‘highly embarrassing’ or ‘cringey’.^20^ Young people wanted privacy^20^ and for SRE to take place in confidence,^20^ ^43^ ^81^ with trustworthy educators,^39^ but teachers were not always trusted to maintain confidentiality^20^ ^39^ ^40^ ^50^ ^51^ ^57^ ^58^ and some doubted their impartiality.^52^ ^57^ Teachers were also perceived to be moralistic,^20^ ^40^ ^50^ to judge students according to different values^40^ ^46^ ^51^ and to have difficulty accepting that students were sexually active.^50^ ^76^ In addition, teacher–student power relations were regarded as problematic,^50^ ^76^ with some students attempting to shift the balance of power^86^ and testing teachers' ability to cope with delivering SRE.^41^

Students reported good, mutually respectful relationships with peer educators,^40^ ^65^ forgiving their occasional lack of preparation^59^ ^87^ or inability to manage disruption.^40^ They felt a sense of affinity with them^53^ ^64^ ^87^ which encouraged them to believe what peer educators told them;^88^ those receiving peer-led SRE reported it had an impact on them, changed their attitudes or taught them something new.^40^ ^65^ ^87^ ^88^ Some felt that peer educators were credible due to their (assumed) sexual experience,^40^ but others felt that their credibility was undermined by youth^58^ ^81^ or lack of knowledge.^53^ ^58^ Nevertheless, students liked their discussion-based approaches.^40^ ^87^ Some peer educators used techniques for creating a safe environment and their lack of relationship with students made them easier to trust than teachers.^40^ However, some students were concerned they might not take confidentiality seriously enough^53^ ^59^ and it was suggested that their looser classroom management style might increase young women's vulnerability.^40^

Many young people liked the idea of sexual health professionals delivering SRE.^20^ ^39^ ^40^ ^50^ ^54–56^ ^81^ Such professionals were perceived to be less judgemental, more informed and better at delivering SRE than teachers,^39^ ^77^ although maintaining discipline was occasionally reported to be problematic.^43^ Some liked the idea of school visits to sexual health clinics^81^ and classes at a school-linked clinic were enthusiastically received by students who appreciated the educator's expertise and enthusiasm.^52^ Outside experts were felt to ‘know what they are talking about’,^53^ provide greater confidentiality^20^ ^40^ and lessen embarrassment^52^ due to their anonymity.^20^ ^37^ ^52^ ^56^ However, some disagreed about whether anonymous or known sex educators were preferable,^53^ with others suggesting both.^43^ Young people's views on the qualities of good sex educators are given in table 6.

**Table 6:** Young people's views on the qualities of a good sex educator

Qualities	Authors
Knowledgeable	Strange *et al*;^47^ Allen;^53^ Kimmel *et al*;^39^ Lupton and Tulloch;^50^ Kanahols *et al*^52^
Has expertise in sexual health	Strange *et al*;^47^ Forrest *et al*;^40^ Abel and Fitzgerald;^74^ O'Higgins and Gabbhain;^55^ Lupton and Tulloch^50^
Professional	Allen;^53^ Lupton and Tulloch;^50^ Kimmel *et al*^39^
Specifically trained in SRE	Dicenso *et al*;^57^ Allen;^53^ Kimmel *et al*^39^
Confident, unembarrassed, straightforward, approachable and unshockable, experienced at talking about sex, uses everyday language	Strange *et al*;^47^ Forrest *et al*;^40^ O'Higgins and Gabbhain;^55^ Eisenberg *et al*;^56^ Kanahols *et al*^52^ Hilton;^43^ Dicenso *et al*^57^
Trustworthy, able to keep information confidential	Kimmel *et al*;^39^ Hilton^43^
Has experiential knowledge, comfortable with own sexuality	Lupton and Tulloch;^50^ Measor *et al*;^42^ Lester and Allan^81^
Good at working with young people	Forrest *et al*;^40^ Allen^53^
Able to relate to and accept young people's sexual activity	Measor *et al*;^42^ Lupton and Tulloch^50^
Respectful of young people and their autonomy, treats them as equals	Selwyn and Powell;^58^ Dicenso *et al*;^57^ Eisenberg *et al*;^56^ Lupton and Tulloch^50^
Has similar values to youth, provides balanced view, non-judgemental	Strange *et al*,^47^ Hilton;^43^ Kimmel *et al*^39^

## Discussion

The synthesis indicates that schools take insufficient account of the ‘specialness' of sex as a topic, negatively affecting the way SRE is delivered and rendering many young people vulnerable and reluctant to engage. The synthesis also suggests that schools struggle to accept that some young people are sexually active, leading to SRE content that is out of touch with many young people's lives. Finally, the evidence indicates that young people dislike having their own teachers deliver SRE.

Despite the wide geographical reach of the studies, young people's views are strikingly consistent. It might be imagined that some studies would produce highly contrasting data, such as the studies from Japan^76^ or Iran,^68^ but this was not the case; Japanese and Iranian students' main concerns reflected those of their peers elsewhere. Similarly, the views of students in the USA who had abstinence-only-based SRE were not markedly different from those whose SRE was more comprehensive; young people's accounts suggest that sex was commonly presented negatively whether their SRE was explicitly abstinence-based or not. Nor did young people's views differ according to whether they had left, or were still in, school. Furthermore, students' views are remarkably consistent over the 25 years spanned by the studies. The four studies published in the 1990s do not particularly stand out, nor do changes in UK policy over the period (eg, the 1998 Teenage Pregnancy Strategy or the 2000 DfE Guidance)^8^ appear to have had an impact on British young people's assessments. The significant recent cultural changes (indicated by equalisation of the age of consent in 2001, Repeal of Section 28 in 2003 and the increasing number of countries introducing same-sex marriage laws) are, however, reflected in young people's views, but what young people express is frustration that these cultural changes are not yet reflected in SRE.

We found no studies of young people's views of SRE that covered issues such as sexting or online safety. While these topics are clearly being discussed within schools,^89^ research into young people's views of such programmes had not been published at the time of our synthesis. Similarly, although many of the studies reported young people's views on inequalities of gender and sexuality within SRE, only one of the studies raised the issue of social class inequalities.^46^ Sexual health is influenced by a wide range of interconnected social and cultural factors (including gender norms and inequities, socioeconomic status, poverty, social exclusion, sexual violence and local legal and policy frameworks,^90^) and despite focusing on SRE, we recognise the influence of these factors. Nevertheless, this is the first time that qualitative studies of young people's views of their SRE have been synthesised; until now the evidence from these geographically disparate studies has been scattered and fragmented. Bringing together students' perspectives in this way helps to give young people a stronger voice^91^ and generates robust evidence. The qualitative nature of the study—and in particular, the meta-ethnographic method that encouraged us towards a fresh interpretation of the data—allows us to understand in much greater depth the reasons for young people's dissatisfaction with SRE, producing the insight necessary for recommending how improvements are to be achieved.

Owing to the ‘specialness' of sex as a topic,^36^ the delivery of SRE requires careful planning; it cannot simply be provided in the same way as other subjects. As Hawkes^36^ observes, sex raises numerous individual and social anxieties about, for example, unregulated sexual desire, female sexuality, the consequences of non-procreative erotic practices and young people's sexuality. Discussions about sex and relationships touch on intimate issues, creating potential for embarrassment which may be particularly acute for adolescents. Schools have adopted a ‘no-nonsense’ approach that asserts that sex is not embarrassing, but we argue that schools have adopted this particular approach precisely because discussing sexual issues can be awkward and embarrassing. A reluctance to acknowledge our continuing anxieties about sex means disregarding the very real difficulties experienced by young people in SRE. Teachers also describe ‘discomfort’ when delivering SRE, reporting that it is ‘fraught with difficulties'.^92^ Only once schools acknowledge that sex is a special topic can SRE be delivered in a way that makes students and educators feel safer. We suggest that key factors include the delivery of SRE by professional sexual health educators who are able to provide excellent class control, as well as single-sex classes at least some of the time.

We suggest that unless schools accept the fact and range of many young people's sexual activity, students will continue to report that SRE content is out of touch and irrelevant and will be less likely to engage in—and so benefit from—their SRE. Schools' reluctance to acknowledge young people's sexual activity may be influenced by the anticipated reactions of parents or the media, or by lack of confidence about what can be taught in SRE. However, our society is anxious about young people's sexuality^1^ ^36^ and teachers are no exception.^92^ (While much of this anxiety appears to relate to young people having sexual intercourse, in fact only a minority of under 16s engage in heterosexual intercourse).^93^ Young people's aspirations for SRE appear to align with a ‘sex-positive’ approach that aims for young people to enjoy their sexuality in a way that is safe, consensual and healthy.^94^ However, even if excellent SRE curriculum materials were produced, the success of those materials would depend in very large part upon the educator delivering them.

Young people's discomfort with the current practice of having SRE delivered by their own teachers appears to represent, among other things, a plea for clear roles and boundaries. This emerging evidence finds support in a recent survey that reveals that only 19% of 18-year olds feel SRE should be taught by a teacher from their school,^21^ as well as elsewhere.^95^ In addition to the issue of blurred boundaries however, teachers may be inherently unsuitable for delivering SRE because of the nature of the student–teacher relationship. This relationship is ideally constructed as desexualised, so discussing sexual issues can be difficult for teachers^50^ and may disrupt attempts to control sexualised behaviour.^96^ Furthermore, the power imbalance inherent in the teacher–student relationship can be problematic and inappropriate within the context of SRE.^95^ ^97^ Hawkes suggests that young people find it difficult to discuss sex and relationships in a context characterised by an imbalance of social power, ‘even when socially sanctioned, as in sex education’ (ref. 36, p. 5). Indeed, one of the theories supporting the delivery of SRE by peer educators is that egalitarian interaction might allow for more open communication about sexual issues.^98^ In contrast, the practice of teacher-led SRE not only lacks theoretical support,^97^ but may even challenge the maintenance of ethical teacher–student boundaries.^99^

Nevertheless there is likely to be considerable resistance to any suggestion that teachers should not deliver SRE. Teachers are regarded as the most sustainable option since they are already embedded in schools and constitute an ongoing resource; outside experts, it is argued, are expensive, while peer educators have to be regularly trained up as existing cohorts age. Perhaps a compromise would be the provision of specialist SRE teachers who only teach SRE and whom students would not encounter except in the context of SRE. This might deal with young people's need for clear boundaries, while also encouraging expertise and professionalism in SRE. The role of specialist SRE teachers could be developed so that it is clearly distinct from that of other teachers, with a view to lessening the traditional teacher–student power imbalance in that context.

There is a need to further explore young people's views about who should deliver SRE—specifically examining issues relating to boundaries, anonymity and power—and whether these views vary with age. Younger children did seem more positive about SRE than older children, but since our synthesis only included one study of primary school children, this evidence is only suggestive, making it difficult to draw any solid conclusions about the significance of age. Research should investigate the acceptability to young people of SRE being delivered by highly trained, specialist teachers who only teach SRE. Unless we get the delivery, right young people will continue to disengage from SRE and opportunities for safeguarding young people and improving their sexual health will be reduced.
