# Supplementary material for: What do young people think about their school-based sex and relationship education? A qualitative synthesis of young people's views and experiences
Source: BMJ Open. 2016 Sep 1;6(9):e011329. doi: 10.1136/bmjopen-2016-011329 (PMC5030567; doi:10.1136/bmjopen-2016-011329)
Supplement: supplementary appendices [file bmjopen-2016-011329supp_appendices.pdf]

Appendix 1: Criteria for quality appraisal

We developed our quality criteria after reviewing the available checklists (see below) for appraising qualitative research, including our own previous adaptation of the CASP criteria:

1. Does the research report on findings from qualitative research and did that research involve both qualitative methods of data collection and analysis?
2. Does the research have clear aims and objectives?
3. Were the data collected in a way that addressed the research aim?
4. Was the data analysis sufficiently rigorous to address the aims of the research?
5. Can one be confident that all the relevant data were taken into account?
6. Were sufficient data presented to support the interpretations made?
7. What was the quality of reporting of the methods?
8. Did the paper demonstrate theoretical insight, novel findings or perspectives?
9. Overall quality assessment

Checklists for appraising qualitative research

- Critical Appraisal Skills Program. (1988) Ten questions to help you make sense of qualitative research. CASP collaboration for qualitative methodologies. http://media.wix.com/ugd/dded87_951541699e9edc71ce66c9bac4734c69.pdf (accessed 11. 2. 14)
- Popay J, Rogers A, Williams G. 1998. Rationale and standards for the systematic review of qualitative literature in health services research. Qualitative Health Research 8(3): 341-351. http://www.qualres.org/HomePopa-3686.html
- Burns N. (1989) Standards for qualitative research. Nursing Science Quarterly 2(1): 44-52.
- Pope C, Mays N. Assessing quality in qualitative research. BMJ 2000; 320: 50-2.
- Spencer L, Ritchie J, Lewis J, Dillon L. (2003) Quality in Qualitative Evaluation: A framework for assessing research evidence. London: Cabinet Office.
- Dixon-Woods M, Shaw R, Agarwal S, Smith J. The problem of appraising qualitative research. (2004) Quality and Safety in Health Care 13: 223-25.
- ©Letts L, Wilkins S, Law M, Steward D, Bosch J, Westmorland M. (2007) Critical Review Form - Qualitative Studies (Version 2.0). McMaster University.
- Kuper A, Lingard L, Levinson W. (2008) Critically appraising qualitative research. British Medical Journal 337: 687-9.
- Harden A, Brunton G, Fletcher A, Oakley A. Teenage pregnancy and social disadvantage: a systematic review integrating trials and qualitative studies. British Medical Journal Oct 2009.
- Campbell R, Pound P, Morgan M, Daker-White G, Britten N, Pill R, Yardley L, Pope C, Donovan J. (2011) Evaluating meta-ethnography: systematic analysis and synthesis of qualitative research. Health Technology Assessment 15(43).
- JBI QARI Critical Appraisal Checklist for Qualitative Research. http://www.joannabriggs.org/assets/docs/sumari/SUMARI-V5-User-guide.pdf

Appendix 2: Excluded papers

Papers excluded on grounds of quality

Aquilino ML, Bragadottir H. Adolescent pregnancy. Teen perspectives on prevention. *Am J Matern Child Nurs*. 2000;225:192-197.

Bourton V. Sex education in school: young people's views. *Paediatr Nurs*. 2006;8(8):20-23.

Cook S. Taking account of what young women want from school sex education: Two groups from Scotland and Uganda. *Journal of Health, Organisation and Management*. 2010;24:528-533.

Reid JM, van Teijlingen ER. Perceptions of school-aged women in northeast Scotland on sex education: a focus group study. *Int J Health Promot Educ*. 2006; 44:59-64.

Smith PB, Realini JP, Buzi RS, Martinez M. Students' experiences and perceived benefits of a sex education curriculum: a qualitative analysis. *J Sex Marital Ther*. 2011;37:270-85.

Bull S, Nabembezi D, Birungi R, Kiwanuka J, Ybarra M. Cyber-Senga: Ugandan youth preferences for content in an internet-delivered comprehensive sexuality education programme. East Afr J Public Health. 2010;7:58-63.

Papers excluded due to insufficient data on synthesis topic

Dudley J, Crowder A, Montgomery TR. Back to basics: how young mothers learn about sex and sexuality. *Sex Educ*. 2014; 14(3): 272-285.

Haglund K. Recommendations for sexuality education for early adolescents. *Journal of Obstetric, Gynaecologic, and Neonatal nursing*. 2006; 35: 369-375.

Halpern-Meekin S. School Variation in Students' Experiences of High School Relationship and Marriage Education Courses. *J Fam Issues*. 2010; 33: 1688-1712.

Ingram J, Salmon D. Young people's use and views of a school-based sexual health drop-in service in areas of high deprivation. *Health Educ J*. 2010; 69: 227-235.

Jones TCK. Exploring adolescent mothers' perceptions of school-based sexuality education. Arkansas, University of Arkansas for Medical Sciences, 2009.

Lees S. Talking about sex in sex education. Gend Educ. 1994;6(3):281-292.

Strange V, Forrest S, Oakley A, the RIPPLE Study team. What influences peer-led sex education in the classroom? A view from the peer educators. *Health Educ Res*. 2002;17(3):339-49.

Vuttanont U, Greenhalgh T, Griffin M, Boynton P. "Smart boys" and "sweet girls". Sex education needs in Thai teenagers: A mixed-method study. *Lancet*. 2006; 368: 2068-2080.

Appendix 3: List of included studies (n = 55)

Abel G, Fitzgerald L. 'When you come to it you feel like a dork asking a guy to put a condom on': is sex education addressing young people's understandings of risk? *Sex Educ*. 2006;6(2):105-119.

Alldred P, David M. *Get real about sex: The politics and practice of sex education*. Maidenhead, Open University Press, 2007.

Allen L. Closing sex education's knowledge/ practice gap: the reconceptualisation of young people's sexual knowledge. *Sex Educ*. 2001;1(2):109-122.

Allen L. 'Pleasurable pedagogy': young people's ideas about teaching 'pleasure' in sexuality education. *21st Century Society*. 2007; 2(3): 249-264.

Allen L. 'They think you shouldn't be having sex anyway': young people's suggestions for improving sexuality education content. *Sexualities*. 2008;11(5):573-594.

Allen L. 'It's not who they are it's what they are like': re-conceptualising sexuality education's 'best educator' debate. *Sex Educ*. 2009;9(1):33-49.

Broadbear JT, Broadbear BC. Online Discussion about Sexuality Education in Schools. *International Electronic Journal of Health Education*. 2012;15:16-28.

Buston K, Wight D, Hart G. Inside the sex education classroom: the importance of context in engaging pupils. *Culture, Health and Sexuality*. 2002;4(3):317-335.

Buston K, Wight D. The salience and utility of school sex education to young women. *Sex Educ*. 2002;2(3):233-250.

Buston K, Wight D. The salience and utility of school sex education to young men. *Sex Educ*. 2006;6(2):135-150.

Castro-Vasquez G, Kishi I. 'Nemureru ko wo okosu mono dearu': learning about sex at a top ranking Japanese Senior High School. *Sexualities*. 2002;5(4):465-486.

Chambers D, Tincknell E, Van Loon J. Peer Regulation of Teenage Sexual Identities. *Gend Educ.* 2004;16:397-415.

DiCenso A, Borthwick VW, Busca CA, Creatura C, Holmes JA, Kalagian WF, Partington BM. Completing the picture: Adolescents talk about what's missing in sexual health services. *Can J Public Health*. 2001;92:35-38.

Eisenberg M, Wagenaar A, Neumark-Sztainer D. Viewpoints of Minnesota students on school-based sexuality education. *J Sch Health*. 1997;67(8):322-326.

Fonseca AD, de Olivera L, Teixeira KC. Perception of adolescents about an educative action in sexual orientation conducted by nursing academics [Portuguese]. *Anna Nery School Journal of Nursing / Escola Anna Nery Revista de Enfermagem.* 2010;14:330-337.

Formby E. Sex and relationships education, sexual health and lesbian, gay and bisexual cultures: views from young people. *Sex Educ*. 2011;11(3):255-266.

Forrest S, Strange V, Oakley A, the RIPPLE Study Team. A comparison of students' evaluations of a peer-delivered sex education programme and teacher-led provision. *Sex Educ*. 2002;2(3):195-214.

Gubrium AC, Shafer MB. Sensual sexuality education with young parenting women. *Health Educ Res*. 2014; doi:10.1093/her/cyu001

Haste P. Low opinions, high hopes: revisiting pupils' expectations of sex and relationship education. *Sex Educ*. 2013;13(5):522-534.

Hilton G. Listening to the boys: English boys' views on the desirable characteristics of teachers of sex education. *Sex Educ*. 2003;3(1):33-45.

Hilton G. Listening to the boys again: an exploration of what boys want to learn in sex education classes and how they want to be taught. *Sex Educ*. 2007;7(2):161-174.

Hirst J. Researching young people's sexuality and learning about sex: experience, need, and sex and relationship education. *Culture, Health and Sexuality*. 2004;6(2):115-129.

Hirst J. Developing sexual competence? Exploring strategies for the provision of effective sexualityies and relationships education. *Sex Educ*. 2008;8(4):399-413.

Hirst J. It's got to be about enjoying yourself': young people, sexual pleasure, and sex and relationships education. *Sex Educ*. 2013;13(4):423-36.

Hyde A, Howlett E, Drennan J, Brady D. Masculinities and young men's sex education needs in Ireland: Problematizing client-centred health promotion approaches. *Health Promot Int*. 2005;20:334-341.

Javadnoori M, Roudsari RL, Hasanpour M, Hazavehei SMM, Taghipour A. Female adolescents' experiences and perceptions regarding sexual health education in Iranian schools: A qualitative content analysis. *Iran J Nurs Midwifery Res.* 2012;17:539-46.

Kanahols AF, Magnusson H, Alehagen S. Swedish adolescents' experiences of educational sessions at Youth Clinics. *Sexual and Reproductive Healthcare*. 2011;2:119-123.

Kidger J. 'You realise it could happen to you': the benefits to pupils of young mothers delivering school sex education. *Sex Educ*. 2004;4(2):185-197.

Kimmel A, Williams TT, Veinot TC, Campbell B, Campbell TR, Valacak M, Kruger DJ. 'I make sure I am safe and I make sure I have myself in every way possible': African-American youth perspectives on sexuality education. *Sex Educ*. 2013;13(2):172-185.

Kubicek K, Beyer WJ, Weiss G, Iverson E, Kipke MD. In the Dark: Young Men's Stories of Sexual Initiation in the Absence of Relevant Sexual Health Information. *Health Educ Behav*. 2010;37:243-263.

Langille D, Mackinnon D, Marshall E, Graham J. So many bricks in the wall: young women in Nova Scotia speak about barriers to school-based sexual health education. *Sex Educ*. 2001;1(3):245-257.

Layzer C, Rosapep L, Barr S. A Peer Education Program: Delivering Highly Reliable Sexual Health Promotion Messages in Schools. *J Adolesc Health*. 2014;54:S70-S77.

Lester C, Allan A. Teenage sexual health needs: asking the consumers. *Health Educ*. 2006;106(4):315-328.

Levin DS. *Let's talk about sex...education: Exploring youth perspectives, implicit messages, and unexamined implications of sex education in schools*. Dissertation Abstracts International: Section B: The Sciences and Engineering. 2010; 71: 3383.

Limmer M. Young men, masculinities and sex education. *Sex Educ*. 2010;10(4) 349-358.

Lupton D, Tulloch J. 'All red in the face': students' views on school-based HIV/AIDS and sexuality education. *Sociol Rev*. 1996;44(2):252-271.

MacDonald J, Gagnon AJ, Mitchell C, Di Meglio G, Cox J. Asking to listen: towards a youth perspective on sexual health education and needs. *Sex Educ*. 2011;11(4):443-457.

McKee A, Watson A-F, Dore J. ‘It's all scientific to me’: focus group insights into why young people do not apply safe-sex knowledge. *Sex Educ*. 2014;14(6):652-65.

Measor L. Young people's views of sex education: gender, information and knowledge. *Sex Educ*. 2004;4(2):153-166.

Measor L, Tiffin C, Miller K. *Young people's views on sex education*. London: Routledge Falmer, 2000.

O'Higgins S, Gabhainn SN. Youth participation in setting the agenda: learning outcomes for sex education in Ireland. *Sex Educ*. 2010;10(4):387-403.

Orgocka A. Perceptions of communication and education about sexuality among Muslim immigrant girls in the US. *Sex Educ.* 2004;4(3):255-271.

Paul G, Bell C, Fitzpatrick A, Smith SM. 'The real deal': A feasibility study of peer-led sex education for early school leavers. *Eur J Contracept Reprod Health Care*. 2010;15:343-356.

Rawson HA, Liamputtong P. Culture and sex education: The acquisition of sexual knowledge for a group of Vietnamese Australian young women. *Ethnicity and Health*. 2010;15:343-364.

Rolston B, Schubotz D, Simpson A. Sex education in Northern Ireland schools: A critical evaluation. *Sex Educ*. 2005;5:217-234.

Rye B, Mashinter C, Meaney GJ, Wood E, Gentile S. Satisfaction with previous sexual health education as a predictor of intentions to pursue further sexual health education. *Sex Educ*. 2015;15(1):93-107.

Selwyn N, Powell E. Sex and relationships education in schools: the views and experiences of young people. *Health Educ*. 2007:107(2):219-231.

Strange V, Oakley A, Forrest S, the RIPPLE Study Team. Mixed-sex or single-sex sex education: how would young people like their sex education and why? *Gend Educ*. 2003;15(2):201-214.

Teitelman AM, Bohinski JM, Boente A. The social context of sexual health and sexual risk for urban adolescent girls in the United States. *Issues Ment Health Nurs*. 2009;30:460-469.

Thomson R, Scott S. *Learning about sex. Young women and the social construction of sexual identity*. (Women Risk and Aids Project. WRAP paper 4). London: The Tufnell Press, 1991.

Tutty LM. Listen to the children: kids' impressions of Who Do You Tell. *J Child Sex Abus*. 2014; 23(1):17-37.

Van Teijlingen E, Reid J, Shucksmith J, Harris F, Philip K, Imamura M, Tucker J, Penney G. Embarrassment as a key emotion in young people talking about sexual health. *Sociological Research Online*. 2007;12.

Woodcock A, Stenner K, Ingham R. 'All these contraceptives, videos and that ...': young people talking about school sex education. *Health Educ Res*. 1992;7(4):517-531.

Yoo S, Johnson CC, Rice J, Manuel P. A qualitative evaluation of the Students of Service (SOS) program for sexual abstinence in Louisiana. *J Sch Health*. 2004;74:329-334.

Yu J. School sex education: views within British-Chinese families. *Asian Journal of Nursing*. 2007;10:171-17.

Appendix 4: Features of the 55 papers (48 studies) synthesised

Author(s)	Study type	Country	Sample	Participants	SRE programme	Data collection
Thomson & Scott 1991	Explores young women's accounts of their sex education	*UK England*	Unreported (not school)	70 females aged 16-21; predominantly white	Sex education	Interviews
Woodcock et al 1992	Explores young people's views of SSHE	*UK England*	Colleges, hostels, youth centres, sports clubs	50 females, 50 males aged 16-25; 97% white European	School sex education	Interviews
Lupton & Tulloch 1996	Explores students' responses to school HIV & sexuality education	*Australia*	5 secondary schools	65 female, 73 male school pupils aged 16-17	School based HIV/ AIDs & sexuality education	Focus groups
Eisenberg et al 1997	Investigates adolescents views of SSHE	*USA*	5 public high schools	24 female, 5 male school pupils aged 14-18; White 20, African American 9	School based sexuality education	Focus groups
Measor et al 2000[b]	Explores young people's views of SSHE	*UK England*	5 comprehensive schools	Total no. & males/ females unreported School pupils aged 13-15	Variety of standard & innovative programmes	Observation; interviews
Langille et al 2001	Investigates barriers to young women receiving effective SSHE	*Canada*	1 high school	28 female school pupils aged 15-18; Euro-Canadian 25, African-Canadian 3	Personal development & relationships	Interviews
DiCenso et al 2001	Explores adolescents' opinions of SSHE	*Canada*	2 high schools	49 female, 34 male school pupils aged 13-19	School sex education	Focus groups
Allen 2001	Explores young people's views of SSHE	*New Zealand*	7 schools	Total no. & males/ females unreported; mainly school pupils aged 17-19; European 57%, Pacific Islands 16%, Maori 16%, Asian 9%	School based sexuality education	Focus groups; interviews
Buston et al 2002[a]	Investigates a conducive atmosphere for delivering SSHE	*UK Scotland*	6 co-educational state schools	78 female, 84 male white school pupils aged 14-16	3 schools standard sex education; 3 SHARE	Interviews; focus groups
Buston & Wight 2002[a]	Explores young women's accounts of the role of SSHE	*UK Scotland*	6 co-educational state schools	78 white, female school pupils aged 14-16	3 schools standard sex education; 3 SHARE	Interviews; focus groups
Forrest et al 2002[b] (RIPPLE)	Compares pupils' views of peer versus teacher-led SSHE	*UK England*	19 comprehensive secondary schools	179 female, 193 male school pupils aged 13-14	14 schools peer-led & 5 teacher-led SRE	Focus groups

Castro-Vasquez & Kishi 2002	Seeks students' views to develop relevant SSHE	*Japan*	1 co-educational senior high school	51 male Japanese school pupils aged 15-18	Japanese school sex education	Interviews
Hilton 2003[a]	Explores boys' preferences regarding the characteristics of sex education teachers	*UK England*	1 mixed comprehensive; 1 boys' private school; 1 boys' grammar	24 male school pupils aged 16-17	School sex education	Focus groups
Strange et al 2003[b] (RIPPLE)	Investigates young people's views about single versus mixed sex SSHE	*UK England*	6 of the 27 mixed sex state schools in RIPPLE	45 female, 45 male school pupils aged 13-15; white except for 6 Asian girls, 1 Indian, 2 Pakistani & 3 African-Caribbean boys	Teacher or peer-led sex education	Focus groups
Hirst 2004[a]	Explores the relevance of SSHE	*UK England*	1 school	11 female, 4 male school pupils aged 15-16; White 6, Somali 4, Pakistani 3, African-Caribbean 2	SRE	Focus groups; interviews
Measor 2004[b]	Explores adolescent sexuality & their views of their SSHE	*UK England*	6 comprehensive schools	Total no. & no. males/ females unreported; School pupils aged 12-15	Sexuality education	Focus groups; interviews; observation
Kidger 2004	Explores pupils' experiences of SSHE given by young mothers	*UK England*	5 comprehensive schools	34 female, 15 male school pupils aged 14-17	*SSHE* delivered by young mothers	Focus groups; interviews
Yoo et al 2004	Evaluates an abstinence only sexual health programme	*USA*	5 schools	32 female, 36 male school pupils, mean age 15; White 52, Black 11, Hispanic 3, Asian 2	Abstinence only programme given by peer mentors	Focus groups
Orgocka 2004	Explores how Muslim girls' sex education is mediated through mothers & SSHE	*USA*	Snowball sampling in mosques & Islamic centres	38 females, mean age 16; school pupils 74%, college 23%, home-schooled 3%; US born Muslims 76%, migrated to US 24%	School based sexuality education classes	Focus groups; interviews
Chambers et al 2004	Explores young people's views about the relevance of SSHE	*UK England*	2 mixed comprehensives; 1 private girl's school; 1 private boy's school	'just over 100' school pupils aged 12-15; No. males/ females unreported; White, Asian, S.E. Asian, African Caribbean	Each school had its own sexual health education curriculum	Focus groups

Rolston et al 2005	Explores young people's opinions about the quality of their SSHE	*UK Northern Ireland*	Schools, youth clubs, universities, etc.	Total no. in focus groups 'around 700' (no. males/ females unreported); interviews with 8 females and 7 males; aged 14-25	School sex education	Focus groups; interviews
Hyde et al 2005	Explores issue of self-defined sex education needs	*Republic of Ireland*	3 mixed & 7 single-sex schools	102 female, 124 male school pupils aged 15-16 & 18-19	School sex education	Focus groups
Buston & Wight 2006[a]	Explores young men's views on their SSHE	*UK Scotland*	6 co-educational state schools	83 male school pupils aged 14-16 White except for 1 Chinese male	3 schools standard sex education; 3 SHARE	Focus groups; interviews
Lester & Allan 2006	Explores young people's views of sexual health services, SSHE, STIs	*UK Wales*	3 comprehensive schools	16 female, 16 male school pupils aged 14-15	School sex education	Focus groups
Abel & Fitzgerald 2006	Explores students' feedback on their SSHE	*New Zealand*	1 mixed secondary school	21 females, 21 male school pupils aged 14-15	Health & Physical Education	Focus groups; interviews
Alldred & David 2007	Explores different perspectives and hopes for SRE in schools	*UK England*	17 secondary schools	161 female, 164 male school pupils aged 13-14; 10 young mothers, 1 young father not in school; 13 young men aged 13-15 not in school	School sex education	Focus groups, interviews, informal discussions
Allen 2007[a]	Explores young people's ideas about incorporating pleasure into SSHE	*New Zealand*	5 secondary schools; GLBT youth community group	81 school pupils, aged 16-19; nos. male/ female & no. in community group unreported	Sexuality education	Focus groups
Hilton 2007[a]	Explores what/ how boys want to learn in SRE classes	*UK England*	1 mixed comprehensive, 1 boarding school, 1 grammar	24 male school pupils aged 16-17; representative of their schools in ethnicity, SES, academic ability	SRE	Focus groups
Selwyn & Powell 2007	Investigates how young people use SRE to obtain information	*UK Wales*	3 schools; 6 other settings (youth clubs, youth events)	20 females, 37 males aged 13-18; White British 37, non-white British 18	Schools all implementing SRE guidelines	Focus groups
Yu 2007	Reports British-Chinese views on SSHE	*UK Scotland*	Language schools, church, women's groups	10 females, 10 males aged 16-19; British born to Chinese parents	School sex education	Interviews

Van Teijlingen et al 2007	Explores embarrassment in relation to discussing sexual health	*UK Scotland*	3 secondary schools	Total no. & no. males/ females unreported; school pupils, aged 12-13 & 16-17	Standard SRE & Healthy Respect intervention	Focus groups
Allen 2008[a]	Explores students' views on relevant SSHE	*New Zealand*	5 secondary schools; GLBT youth group	78 participants aged 16-19; nos. male/ female unreported, mainly school pupils	Sexuality education	Focus groups
Hirst 2008[a]	Explores factors relating to sexual competence	*UK England*	1 secondary school	11 female, 4 male school pupils aged 15-16; Pakistani 3, Somali 4, White 6, African-Caribbean 2	School based sexualities & relationships education	Focus groups; interviews
Allen 2009[a]	Explores who young people think make best sex educators	*New Zealand*	5 secondary schools; GLBT youth group	78 participants aged 16-18; no. male/ female unreported, , mainly school pupils	Sexuality education	Focus groups
Teitelman et al 2009	Explores girls' views on how they learn about sex & relationships	*USA*	Primarily through adolescent health centre	33 females aged 14-18; African-American 15, European-American 15, mixed race 3	Sexuality education	Interviews
Levin 2010	Explores young people's experiences of SSHE & its effects	*USA*	University	17 female, 17 male 1st year undergraduates aged 18-20; White/ European 23, Black/ African American 4, East Asian 4, S. Asian, Latino, Arab 3	Comprehensive, abstinence only, or combination of the two	Focus groups
Rawson & Liamputtong 2010	Explores how Vietnamese Australian young women acquire sexual knowledge	*Australia*	Snowball sampling: key informants facilitated access to the community	15 females aged 18-25; 2nd generation Vietnamese Australian; college students 10, unemployed 5	School sex education	Interviews
Paul et al 2010	Explores young women's experience of peer-led sex education by teenage mothers	*Republic of Ireland*	4 community educational training centres	45 female early school leavers aged 16-21	Peer sexual health education delivered by teenage mothers	Focus groups
O'Higgins & Gabhainn 2010	Describes youth input into sexual health curriculum	*Republic of Ireland*	13 schools	248 females, 146 male school pupils aged 15-18	Development of a new sexual health curriculum	Workshops

Limmer 2010	Investigates young men's views on the barriers to effective SRE	*UK England*	Deprived / affluent areas & academic achievers	45 white British males	SRE	Focus groups; interviews
Fonseca et al 2010	Investigates students' views of a peer-led school sexual health project	*Brazil*	1 high school	6 female, 9 male Brazilian school pupils aged 15-17	Peer- led Health & Prevention in Schools Project	Interviews
Kubicek 2010	Explores how young men who have sex with men gain their sexual health education	*USA*	Bars, clubs, street corners & special events	58 males; 74% aged 18-21, 26% 22+; 49% school pupils; African American 19, Caucasian 20, Mexican 19	Sexual education	Interviews
Broadbear & Broadbear 2011	Analyses students' perceptions of school sexuality education	*USA*	Midwestern university	115 female, 52 male undergraduates; 93% aged 18-25	Sexuality education	Online discussions
Macdonald et al 2011	Describes youth input into sexual health curriculum	*Canada*	2 senior high schools	17 female, 13 male school pupils aged 15-17	Student input into new sexual health curriculum	Focus groups
Kanahols et al 2011	Explores pupils' experiences of SRE at Swedish Youth Clinics	*Sweden*	4 secondary schools	15 female, 14 male Swedish school pupils aged 14-16	SRE session at Swedish Youth Clinic	Focus groups
Formby 2011	Gathers views on SRE from viewpoint of lesbian, gay or bisexual young people	*UK England*	Schools, colleges, young people's services & group from LGBT support group	No. males/ females unreported; City 1: 32 people aged 13-23, ethnic mix; City 2: 'small number' young men aged <35, mostly white	SRE	Focus groups; interviews
Javadnoori et al 2012	Explores Iranian girls' views of SSHE	*Iran*	8 high schools	57 Iranian female school pupils aged 14-18	Sexual health education	Focus groups; interviews
Hirst 2013[a]	Explores the importance of pleasure & positive SRE to sexual health	*UK England*	1 secondary school	11 female, 4 male school pupils aged 15-16; Pakistani 3, Somali 4, African-Caribbean 2, White 6	SRE	Focus groups; interviews
Haste 2013	Questions whether low assessments of SRE reflects poor teaching	*UK England*	3 secondary schools	6 female school pupils aged 13-14	SRE	Focus group
Kimmel et al 2013	Explores young people's views of school & community based sexual	*USA*	Community groups, youth venues, study advertised on local radio	39 females, 9 males, mean age 17; Black/ African-American 96%;	School based sexuality education (abstinence until marriage)	Focus groups

	health education			Predominantly students		
Layzer et al 2014	Describes pupils' views of peer-led SSHE	*USA*	4 high schools	60 school pupils aged 14 No. males/ females unreported	'TeenPEP' , i.e. peer-led sexual health workshops	Focus groups; interviews; observation
Tutty 2014	Explores pupils' views of the 'Who do you tell?' programme	*Canada*	2 elementary schools	65 female, 51 male school pupils, aged 6-12; Caucasian 97, East Indian/ Middle Eastern 11, Asian 7, South America 1	'Who do you tell?' A child sexual abuse prevention programme	Focus groups
Mckee et al 2014	Explores pupils' views of SRE in context of safer sex knowledge	*Australia*	5 schools	89 school pupils aged 14-16, no. males/ females unreported	SRE	Focus groups
Gubrium & Shafer 2014	Young mothers' views of a pilot sex education programme	*USA*	An education centre for young mothers	10 females	Pilot sex-positive, sensual sex education programme	Focus group
Rye et al 2015	Explores views of school SRE & understandings of sexual self	*Canada*	University	10 female, 1 male 1st year undergraduates, aged 18-19; White 10, Egyptian 1	SRE	Interviews

SSHE: School sexual health education / SRE: Sex and Relationships Education / SHARE: Sexual Health and Relationships Education

[a] Indicates where more than one paper relates to a single study

[b] Indicates where papers relate to the same study, though not necessarily the same samples
